# Seeing the Song: Left Auditory Structures May Track Auditory-Visual Dynamic Alignment

**DOI:** 10.1371/journal.pone.0077201

**Published:** 2013-10-23

**Authors:** Julia A. Mossbridge, Marcia Grabowecky, Satoru Suzuki

**Affiliations:** 1 Department of Psychology, Northwestern University, Evanston, Illinois, United States of America; 2 Interdepartmental Neuroscience Program, Northwestern University, Evanston, Illinois, United States of America; University of Leicester, United Kingdom

## Abstract

Auditory and visual signals generated by a single source tend to be temporally correlated, such as the synchronous sounds of footsteps and the limb movements of a walker. Continuous tracking and comparison of the dynamics of auditory-visual streams is thus useful for the perceptual binding of information arising from a common source. Although language-related mechanisms have been implicated in the tracking of speech-related auditory-visual signals (e.g., speech sounds and lip movements), it is not well known what sensory mechanisms generally track ongoing auditory-visual synchrony for non-speech signals in a complex auditory-visual environment. To begin to address this question, we used music and visual displays that varied in the dynamics of multiple features (e.g., auditory loudness and pitch; visual luminance, color, size, motion, and organization) across multiple time scales. Auditory activity (monitored using auditory steady-state responses, ASSR) was selectively reduced in the left hemisphere when the music and dynamic visual displays were temporally misaligned. Importantly, ASSR was not affected when attentional engagement with the music was reduced, or when visual displays presented dynamics clearly dissimilar to the music. These results appear to suggest that left-lateralized auditory mechanisms are sensitive to auditory-visual temporal alignment, but perhaps only when the dynamics of auditory and visual streams are similar. These mechanisms may contribute to correct auditory-visual binding in a busy sensory environment.

## Introduction

The detection, localization, and identification of a visual stimulus are facilitated by a simultaneously presented sound that is spatially coincident or normatively associated *(e.g.*, a “meow” sound for a cat target) with the visual stimulus (*e.g.,* [1]–[10]). Such facilitation reflects an interaction between and/or an integration of auditory and visual representations for locations and objects, potentially mediated by the location-based multisensory responses in subcortical structures such as superior colliculus, cross-modal influences on the responses of primary sensory cortical areas, as well as the location- and object-based multisensory responses in higher cortical areas such as lateral occipital-temporal cortex and superior temporal sulcus (*e.g.,* [11]–[19]).

In addition to location- and object-based interactions between coincident auditory and visual stimuli, continuous auditory and visual streams interact based on dynamic congruence. These dynamic interactions have been studied predominantly for speech perception. For example, presentation of visual lip movements facilitates the perception of congruent auditory speech (*e.g.,* [20], [21], [22]). This facilitation is likely mediated by the left superior temporal sulcus (STS), as the left STS is activated disproportionately strongly by a congruent combination of lip movements and speech sounds (*e.g.,* [23], [24]). Cross-modally congruent lip movements and speech sounds also provide a strong cue for perceptual binding. Such binding is helpful for knowing who is saying what when multiple talkers are present, and it is evident in the ventriloquist effect [25].

These facilitative effects of auditory-visual dynamic congruence during speech perception indicate that auditory and visual streams are integrated in the brain, at least when they share similar dynamics. Thus, it is plausible that, even for non-speech stimuli, some general perceptual mechanisms track the temporal alignment of auditory-visual dynamics. Anecdotally, we enjoy watching dancers glide and leap in temporal alignment with music at a concert, but we are displeased when images are misaligned with the sounds due to a transmission delay while watching live footage of an unfolding event on the news. Temporal alignment of auditory-visual dynamics is also likely to provide a general cue indicating that the two streams arise from a common source. For example, temporally aligned limb movements and footsteps often indicate that both dynamic signals originate from a single walker.

The neural substrates underlying the continuous tracking of auditory-visual synchrony, outside the domain of speech perception, are not well understood. Several studies suggest that left auditory cortex is specialized for processing rapidly varying features in sounds (*e.g.,* [26]–[29]). Given that the activity of auditory cortex is influenced by visual signals (see [16] for a review), we hypothesized that left-lateralized auditory mechanisms might extend their specialization in the processing of rapidly varying auditory features to the processing of dynamic associations between rapidly changing auditory and visual features.

Support for this idea arises from a recent study examining an electroencephalographic (EEG) correlate of auditory-visual dynamic congruence using simple periodic stimuli, with the rates of auditory and visual modulation (2.1 or 2.4 Hz) either being identical (congruent condition) or different (incongruent condition) [30]. Additionally, the visual stimuli were flickered at 10 Hz and the auditory stimuli were amplitude-modulated at 11 Hz to track the evoked visual (known as steady-state visual-evoked potential, or SSVEP) and auditory (known as auditory steady-state response, or ASSR) responses that were phase-locked to the respective stimulus modulation frequencies. Stimulus-phase-locked SSVEP and ASSR allow for monitoring of cross-modal influences on visual and auditory sensory responses separately from the concurrent neural activity reflecting cognitive and affective responses to the stimuli (*e.g.,* activity that is not phase-locked to stimulus modulations). Nozaradan et al. [30] showed that both visual and auditory responses were reduced in the incongruent condition relative to the congruent condition. Relevant to the current study, a topographic plot shown in their Figure 3 hints at the possibility that the decrement in the auditory response was stronger in the left hemisphere.

**Figure 3:**
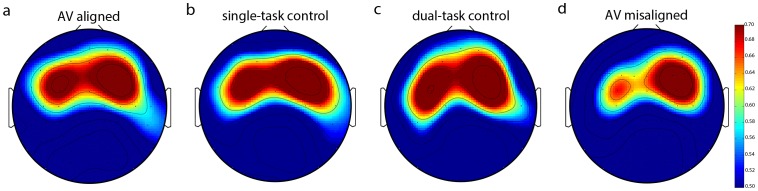
Scalp topography of group average ASSR amplitudes in the (a) AV-aligned, (b) single-task control, (c) dual-task control, and (d) AV-misaligned conditions. Note that ASSR in the left-frontal ROI is reduced only in the AV-misaligned condition (d). Bar to the right indicates scale.

In the present experiments, we made several modifications to Nozaradan *et al.’s* [30] study. Notably, we used complex auditory-visual stimuli relevant to experiences in the real world, classical music combined with a professionally designed music visualizer (iTunes *Jelly*). The music varied in loudness and pitch, while the visualizer generated dynamic visual displays that varied multiple visual features including luminance, color, size, motion, and organization. Furthermore, the music included complex organization across multiple time scales (*e.g.,* rhythmic beats as well as longer variations such as sweeping crescendos), similar to complex auditory stimuli in a natural environment. Our intent was to test the hypothesis that left-lateralized auditory mechanisms track auditory-visual dynamic alignment in a complex environment where cross-modal synchrony is defined by multiple time scales in the context of multiple concurrently varying features. A drawback of this approach is that it would be difficult to differentiate the specific contributions from individual features and time scales. Nevertheless, our use of more naturalistic stimuli complements much research on cross-modal processing focusing on simple periodic stimuli.

An exception is a recent magnetoencephalography (MEG) study that showed that when people watched a feature movie with its own soundtrack, the phase coherence of oscillatory activity in the delta and theta frequencies increased within and across auditory and visual areas as compared to when they watched the same movie while listening to a soundtrack from a different movie [31]. Increased phase coherence while viewing the movie with the congruent soundtrack, however, might reflect auditory-visual semantic congruence instead of cross-modal dynamic alignment, and might also reflect increased cognitive and emotional (rather than perceptual) responses to the movie when it was experienced with its own soundtrack. By using complex auditory-visual stimuli with no semantic associations and by monitoring stimulus-phase-locked auditory responses, here we investigated the perceptual tracking of complex auditory-visual dynamic congruence, separately from cognitive and emotional responses.

As in Nozaradan *et al.* [30], we amplitude-modulated the music to monitor sound-evoked neural activity arising from auditory structures. However, we used a much faster rate of amplitude modulation (40 Hz rather than 11 Hz) so that music was not substantially distorted; the experience was similar to listening to music through an electrical window fan. It is well established that 40-Hz amplitude modulation produces robust phase-locked ASSR that reflects neural activity in subcortical and cortical auditory areas including the primary auditory cortex and surrounding auditory association areas (*e.g.,* [32]–[34]). We also applied a current-source-density (CSD) transformation to our EEG data in order to analyze the topography of ASSR with increased spatial resolution (due to reduced influences from volume conduction) for identifying lateralized neural sources (see the *Methods* section for details).

The visualizer display was either synchronized with the music (the *auditory-visual- [AV-] aligned* condition) or delayed relative to the music (the *AV-misaligned* condition). Based on our hypothesis that left-lateralized auditory mechanisms track auditory-visual dynamic alignment in a complex environment, we predicted that ASSR arising from left (but not right) auditory structures would be significantly larger in the AV-aligned condition than in the AV-misaligned condition.

Such a difference, however, could reflect a difference in attentional engagement with the music; people may be more strongly engaged with the music in the AV-aligned condition when the visual display pleasantly matches the music than in the AV-misaligned condition when the visual display appears disjointed from the music. Some studies have demonstrated that ASSR can be increased with greater auditory engagement [35]–[37], though such top-down modulation of ASSR does not occur in all cases (*e.g.,* [38], [39]). To rule out possible effects of auditory disengagement, we additionally examined ASSR in two control conditions. In a *single-task* control condition, participants were instructed to ignore the music while they attempted to detect a target word presented among a series of sequentially presented words. In a *dual-task* control condition, we further diverted attentional resources from the music by presenting the words in the order of a complex story and instructing participants to detect a target word as well as concurrently follow and remember the story in preparation for a comprehension post-test. Importantly, in both control conditions, the dynamics of word presentation were clearly different than the dynamics of the music.

Given this approach, we reasoned that if attentional engagement with the music drives ASSR, ASSR should be largest in the AV-aligned condition in which a synchronized visualizer display would increase engagement with the music. ASSR should be reduced in the AV-misaligned condition in which a misaligned visualizer display would reduce engagement with the music, but ASSR should be most strongly reduced in the control conditions in which participants actively ignore the music to perform reading tasks, especially in the dual-task control condition in which attentional resources were most diverted from the music.

In contrast, our working hypothesis is that left-lateralized auditory mechanisms monitor the temporal alignment between auditory and visual dynamics irrespective of attentional engagement. Note that auditory-visual temporal alignment is meaningful only when auditory and visual dynamics are similar. We thus reasoned that the responses of left-lateralized auditory mechanisms to music, as probed via ASSR, would not be affected by reading text in the single- and dual-task control conditions because the dynamics of word presentations in those conditions are unrelated to the dynamics of the music. In this sense, the single- and dual-task control conditions provide an auditory-visual baseline that is common in everyday experience, such as listening to music while reading e-mails on a computer screen. Because we hypothesize that left-lateralized auditory mechanisms track auditory-visual temporal alignment irrespective of attentional engagement, we predict that ASSR would be equivalent in the single- and dual-task control conditions. When processing dynamically aligned auditory-visual signals, left-lateralized auditory mechanisms might increase activity (relative to the control conditions) because temporally aligned auditory-visual signals provide consistent information about a common source. In contrast, left-lateralized auditory mechanisms should reduce activity when processing dynamically similar but temporally misaligned auditory-visual signals (based on [30]; see above). Such dynamic misalignment is likely to arise in a busy sensory environment with multiple sources generating similar dynamics, and in this case temporal misalignment would indicate that the attended visual stream is mistakenly bound to the incorrect auditory stream. For example, at a busy party auditory-visual temporal misalignment would indicate that your perceptual system mistakenly paired the mouth movements of a talker you are facing with the voice of another; in this case reducing auditory responses to the wrong voice could facilitate the process of pairing the mouth movements with the correct voice.

In summary, we hypothesize that left-lateralized auditory mechanisms, known to process rapidly varying auditory features, also process temporal alignment between auditory and visual signals when their dynamics are similar. This predicts that left-lateralized ASSR should be largest in the AV-aligned condition, equivalently large or reduced in the two control conditions, and substantially reduced in the AV-misaligned condition. However, if the conditions instead influence ASSR based on the modulation of attentional engagement with the music, left-lateralized ASSR should be most reduced in the two control conditions, especially in the dual-task control condition, in which attention was strongly diverged from the music.

## Materials and Methods

### 2.1 Ethics Statement

The experiments and consent forms were approved by Northwestern University Institutional Review Board (NUIRB). Written informed consent was obtained from each participant, and all investigations were conducted according to the principles expressed in the Declaration of Helsinki.

### 2.2 Participants

Twenty-eight (17 female) right-handed adults (18–29 y.o.) with normal hearing and normal or corrected-to-normal vision responded to a posting on the Northwestern University campus (Evanston, IL, USA), and received monetary compensation for their participation.

### 2.3 Stimuli

Each participant was seated in a comfortable armchair (to reduce muscle artifacts in EEG signals) at 120 cm from the display monitor (21′′, 1024×768 resolution, 60-Hz refresh rate). A six-minute MP3 recording of Beethoven’s *Moonlight Sonata* was sinusoidally amplitude modulated (100%) at 40 Hz and presented over Sennheiser pro headphones at an average level of 70 dB SPL(A). For conditions with longer durations than the recording (the single- and dual-task control conditions), the recording was repeatedly presented throughout the duration of each condition. Amplitude modulation at 40 Hz created a constant 40-Hz buzz as if listening to the music through an electrical window fan, but the music itself could be clearly heard above this droning sound. Video examples of the stimuli used in each of the four conditions (AV-aligned, AV-misaligned, single-task control, and dual-task control conditions) are available online (see https://www.dropbox.com/sh/yrlwfu96qyhum6c/73efhFYfjI).

#### 2.3.1 AV-aligned and AV-misaligned conditions

Each participant listened to an identical 2-minute portion of Beethoven’s *Moonlight Sonata* twice, once while watching the iTunes *Jelly* visualizer display presented in synchrony with the music (the AV-aligned condition), and once while watching the visualizer display presented with a 30-s delay (the AV-misaligned condition). Note that in both conditions, participants heard the same first 2 minutes of the music. Because the music contained a constant beat as well as dynamic variations at longer time scales, the relatively long (30-s) visual delay in the AV-misaligned condition allowed us to introduce substantial asynchrony across multiple time scales (except for coincidental alignments in auditory-visual variations). The visualizer display was presented against a black background with an average luminance of 4.9 cd/m^2^.

In the AV-aligned condition, changes in the luminosity of the features within the visualizer display matched changes in the intensity of the music (see below for verification). Other visual features such as color, speed, motion, and organization also dynamically changed seemingly in synchrony with the changes in auditory intensity and pitch, but these instances of auditory-visual synchronization were less apparent. We emphasize that our goal was to determine the role of left-lateralized auditory mechanisms in tracking auditory-visual alignment in complex naturalistic stimuli that included dynamics across multiple time scales in the context of multiple concurrently varying features, rather than to investigate the processing of a specific combination of features within a specific time scale as in [30]. To avoid potential stimulus artifacts, we freshly generated a new visualizer display for each condition for each participant. Because the visualizer display was different each time in feature specific details, we were able to conservatively distinguish between auditory responses to cross-modal dynamic alignment and misalignment relatively independently of stimulus-specific contributions. Finally, because the visualizer display was presented on a CRT monitor at a refresh rate of 60 Hz, the visual stimuli did not evoke any phase-locked 40-Hz EEG oscillations, so that any effect of auditory-visual alignment on the 40-Hz ASSR indicated cross-modal neural interactions between auditory and visual processes.

Participants were asked to attend to the music and the visualizer display, and to simply enjoy their listening/watching experience. They were not informed that the visualizer display was temporally aligned with the music in one condition and misaligned in the other. They were merely told that they would be watching two 2-minute instances of the same song with different visualizer displays. The stimuli (auditory and visual) were presented with a MacBook Pro laptop computer running OS 10.6.

#### 2.3.2 Visualizer control experiments

The iTunes *Jelly* visualizer is designed to generate visual images that synchronize with music in an aesthetically pleasing manner. However, because it is proprietary software and we had no access to its algorithm, it was necessary to verify that the feature variations generated by the visualizer indeed synchronized with the music in the AV-aligned condition. Specifically, we verified (1) that most people were able to perceptually distinguish the AV-aligned condition from the AV-misaligned condition, and (2) that the temporal correlation between the overall variation in visual luminance and the overall variation in auditory intensity was indeed reliably higher in the AV-aligned condition than in the AV-misaligned condition (see *Text S1*).

#### 2.3.3 Single-task and dual-task control conditions

The single- and dual-task control conditions were included because any reduction in ASSR in the AV-misaligned condition could potentially be due to attentional disengagement from the music when the visual display appeared misaligned with the music. In these control conditions, we presented participants with the same amplitude-modulated music as in the AV-aligned and AV-misaligned conditions, but we told participants to ignore the music and perform a reading task. Reading material consisted of the first 1,182 words of English text from the first chapter of *Doctor Pascal* by Emile Zola. The words (presented with accompanying punctuation marks) were white, their vertical and horizontal visual angles ranged from 0.49 to 0.73° and 0.61 to 3.64°, respectively, and were centrally presented on a black background (4.9 cd/m^2^). Each word was presented for 300 ms with an inter-stimulus-interval (ISI) of 200 ms. The rate of word presentation did not correspond to the primary beat of the music (∼1 beat per 1200 ms on average). In both control conditions, we reduced participants’ engagement with the music by asking them to ignore it and requiring them to perform at least one task.

In the single-task control condition, participants viewed the words from the story presented in a randomized order and were asked to press a button when they saw a target word, “and.” In the dual-task control condition, in addition to asking participants to ignore the music and respond every time “and” was displayed, we presented the same set of words in the order of the original text and instructed participants to follow the story in preparation for a comprehension post-test. The comprehension test, consisting of 16 yes-no questions, was given immediately after the dual-task control condition. None of the participants had read the text previous to the experiment, but they all scored above chance (mean = 14/16), indicating that they made an effort to comprehend the story. Thus, in the dual-task control condition, we further reduced auditory engagement with the music by imposing a greater cognitive and working-memory load than in the single-task control condition.

Each control condition lasted about 10 minutes. Presentation software (version 11.0, Build 04.25.07, www.neurobs.com) was used to present stimuli and to record responses.

### 2.4 Procedure

The single- and dual-task control conditions were run before the AV-aligned and AV-misaligned conditions to avoid drawing attention to the music before it was time for participants to attend to the music. We note that there was no significant order effect for ASSR amplitudes in our ROI in either hemisphere (see below) across the four conditions (main effect of order *F* [3,81] = 1.544, *p*>0.209). The order of the single- and dual-task control conditions and that of the AV-aligned and AV-misaligned conditions were both counterbalanced across participants.

### 2.5 EEG Recording and Analysis

During each condition, EEG was continuously recorded using a 64-channel (10–20 configuration) Biosemi system with a nose reference as well as additional electrodes, one placed lateral to each eye for recording horizontal electro-oculographic (EOG) activity and one placed under the left eye for recording vertical EOG activity, including blinks. Data were sampled at 1024 Hz and bandpass filtered between 0.1 and 100 Hz. The resulting EEG data were segmented into 1-s epochs; epochs with eye blinks and muscle artifacts were manually removed based on vertical EOG activity (generally >100 µV, but adjusted for several participants as necessary), and epochs with saccades were manually removed based on horizontal EOG activity (>100 µV, but adjusted as necessary). The first 80 artifact-free epochs from each participant for each condition were transformed into CSD (Current Source Density) maps using CSDtoolbox Version 1.1 (http://psychophysiology.cpmc.columbia.edu/Software/CSDtoolbox) to obtain a reference-free and high-spatial-resolution measure of EEG signals [40]. Note that, although the control conditions lasted longer than the AV-aligned and AV-misaligned conditions (because a substantial number of words needed to be presented for the comprehension test to be meaningful), we analyzed the EEG data from only the initial 80 clean epochs during which participants listened to the same portion of the music. We used these CSD-transformed EEG waveforms to calculate (1) ASSR amplitude, (2) ASSR phase-locking, and (3) non-stimulus-locked oscillatory neural activity.

#### 2.5.1 ASSR amplitude

ASSR amplitude was computed (for each electrode and each participant) by averaging the EEG waveforms across the 80 epochs, taking a Fast-Fourier Transform (FFT) of the average waveform (using Matlab 7.4.0; Mathworks), then extracting the amplitude of the Fourier component at 40 Hz (at 1-Hz resolution). Averaging the EEG waveforms across the 80 epochs before taking a FFT reduced any contributions from non-phase-locked responses, thus isolating the stimulus-evoked auditory neural responses. CSD-transformed EEG signals offer a conservative estimate of the locations of the underlying neural generators [41], and lateralized CSD-transformed EEG signals in particular reflect the activity of sources that can be reasonably assumed to be located on the same side of the brain (*e.g.,* [42]–[44]). Further, source localization results from EEG, MEG (magnetoencephalography), and PET (positron emission tomography) studies suggest that ASSR evoked by 40-Hz amplitude modulation arises from primary auditory cortex with additional contributions from subcortical structures and auditory association areas including the superior temporal plane [32], [33], [45]–[47]. Note that because EEG signals from subcortical structures are not lateralized on the scalp, a lateralized modulation of ASSR can be reasonably attributed to a modulation of cortical auditory sensory activity in the same side of the brain.

#### 2.5.2 ASSR phase-locking

ASSR phase-locking was computed for each electrode and each participant by taking an FFT of the EEG waveform from each epoch, extracting the complex Fourier coefficient for the 40-Hz component, normalizing it by dividing by its amplitude, averaging these normalized complex coefficients across the 80 epochs, then taking the amplitude of the resultant complex number. This phase-locking measure is commonly referred to as inter-trial phase coherence or ITPC (*e.g.,* [48]), with 0 indicating no phase-locking and 1 indicating perfect phase-locking with the auditory amplitude modulation.

#### 2.5.3 Non-stimulus-locked oscillatory neural activity

Oscillatory neural activity that was not phase-locked to the amplitude modulation of the music was computed for each electrode and each participant by taking an FFT of the EEG waveform for each epoch, averaging the Fourier amplitudes across the non-driven frequencies (±10 Hz relative to the driven frequency, 40 Hz), then averaging these mean amplitudes across the 80 epochs. The choice of averaging Fourier amplitudes over ±10 Hz around the driven frequency was made somewhat arbitrarily because we had no hypothesis about what frequency range should be influenced by auditory-visual dynamic alignment. Nevertheless, using a larger (±40 Hz) or smaller (±5 Hz) frequency range did not affect the statistical analyses presented in the *Results* section. Because Fourier amplitudes were computed for each EEG waveform before averaging across the 80 epochs, non-phase-locked (i.e., stimulus-induced) activity survived the averaging process. Because we excluded the 40-Hz component, which included the response to the 40-Hz amplitude-modulated music, the resulting average amplitude reflected non-stimulus-locked oscillatory neural activity potentially induced by high-level processing of music, including cognitive and emotional responses.

#### 2.5.4 Regions of Interest

**Figure 1:**
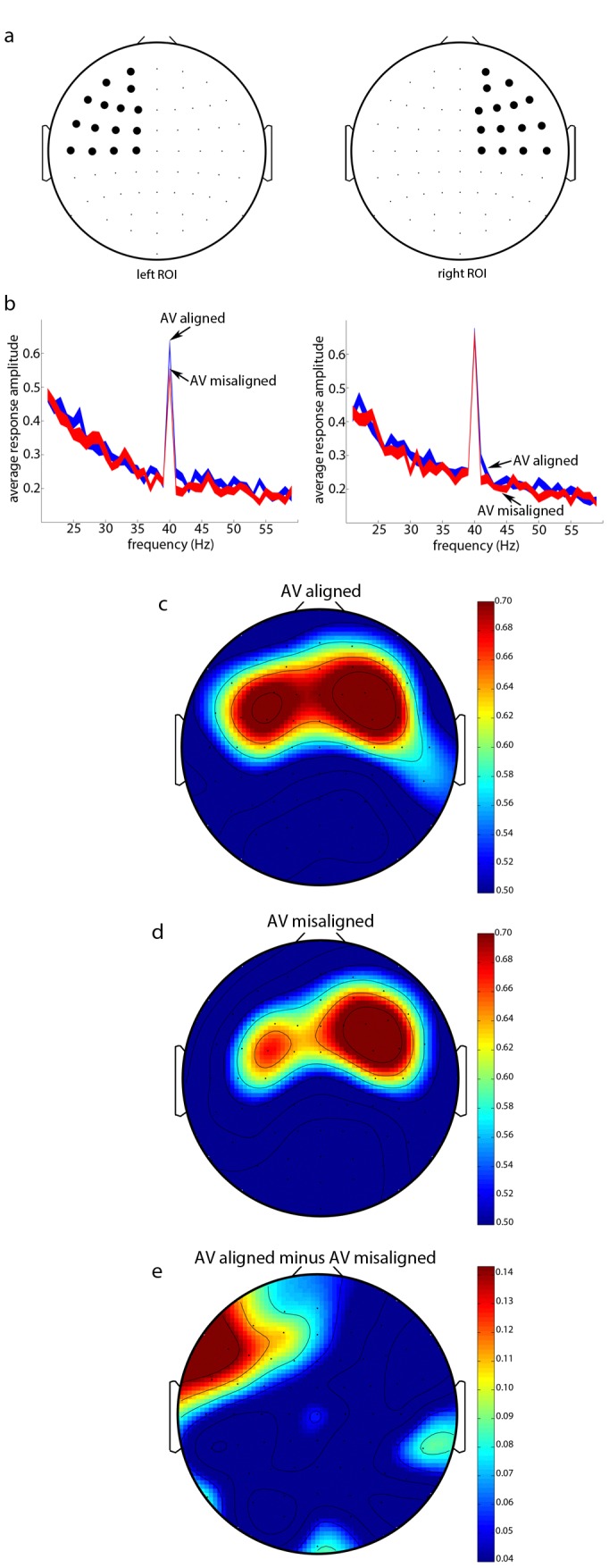
Group average ASSR amplitudes (N = 28) to 40-Hz amplitude-modulated music while participants saw a dynamically aligned or misaligned visualizer. ASSR amplitudes were calculated from current-source density (CSD) transformed EEG scalp potentials. (**a**) Schematics showing the scalp sites comprising the left-frontal ROI (left) and the right-frontal ROI (right). (**b**) Fourier amplitudes shown in 1-Hz resolution, where the 40-Hz peaks correspond to the stimulus-evoked ASSR in the AV-aligned (blue) and AV-misaligned (red) conditions in the left-frontal ROI (left) and the right-frontal ROI (right). Line widths represent ±1 standard error of the mean (s.e.m.), corrected for within-participant comparisons. (**c–d**) Scalp topography of the group average ASSR amplitudes in the AV-aligned (**c**) and AV-misaligned (**d**) conditions. Bars to the right indicate scale. (**e**) Scalp topography of ASSR amplitude difference between the two conditions (AV-aligned – AV-misaligned). Bar to the right indicates scale.

Stimulus-evoked ASSR, ITPC (phase-locking), and stimulus-induced activity were averaged over the scalp sites within the lateralized regions of interest (ROIs). The ROI’s were determined by generating a topographic map of ASSR (averaged across the AV-aligned and AV-misaligned conditions) and selecting the 30 most responsive electrodes, which included 15 electrodes in each hemisphere, yielding the left-frontal and right-frontal ROI’s (see Figure 1a). These ROI’s are similar to the current-source density map for 39 Hz ASSR reported earlier [33].

## Results

ASSR in the left-frontal ROI (Figure 1a, left; see section 2.5) was significantly greater when the visualizer display was dynamically aligned with the music than when it was misaligned (Figure 1b, left), *t*(27) = 2.94, *p*<0.007. In contrast, ASSR in the right-frontal ROI (Figure 1a, right) was unaffected by cross-modal dynamic alignment (Figure 1b, right; *t* [27] = 0.226, *n.s*.). The topographic plots for the auditory-evoked ASSR in the AV-aligned (Figure 1c) and AV-misaligned (Figure 1d) conditions (aligned minus misaligned shown in Figure 1e) clearly indicate that the processing of auditory-visual dynamic alignment is lateralized to the left hemisphere, confirmed by the significant hemisphere by alignment interaction, *F*(1,27) = 4.947, *p*<0.035.

Auditory-visual dynamic alignment had little effect on the degree of phase-locking at 40 Hz in either ROI (ITPC = 0.318 [standard error of the mean; s.e.m. = 0.027] vs. 0.299 [s.e.m. = 0.024] in the AV-aligned vs. AV-misaligned conditions, *t*(27) = 0.680 *n.s.*, for the left-frontal ROI, and 0.361 [s.e.m. = 0.029] vs. 0.353 [s.e.m. = 0.024], *t*(27) = 0.207, *n.s.*, for the right-frontal ROI; see section 2.5.2). This suggests that the left-lateralized decrement in ASSR in the AV-misaligned condition does not reflect a decrease in the temporal precision of the neural response to the music. Further, auditory-visual dynamic alignment did not influence non-stimulus-locked oscillatory neural activity (see section 2.5.3) that could be tied to cognitive and emotional responses to music (average amplitude = 2.14 µv/m^2^ [s.e.m. = 0.189] vs. 1.99 µv/m^2^ [s.e.m. = 0.109] in the AV-aligned vs. AV-misaligned conditions, *t*(27) = 0.377 *n.s.*, for the left-frontal ROI, and 2.05 µv/m^2^ [s.e.m. = 0.109] vs. 2.02 µv/m^2^ [s.e.m. = 0.092], *t*(27) = 0.823 *n.s.*, for the right-frontal ROI). Thus, auditory-visual misalignment reduced the amplitude of auditory neural responses selectively in the left hemisphere without affecting either the fidelity (phase-locking) of these responses or concurrent on-going (non-stimulus-locked) oscillatory neural activity.

**Figure 2:**
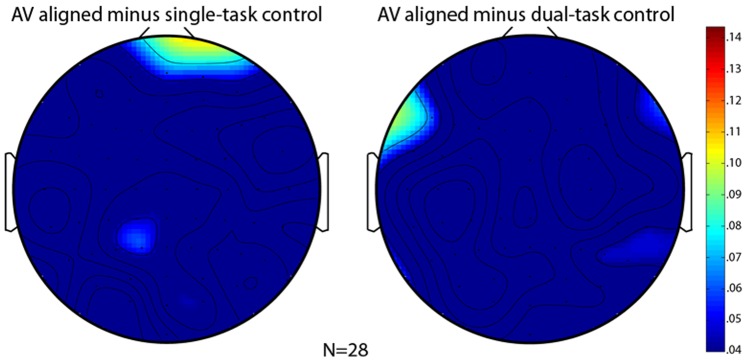
Scalp topography of the ASSR amplitude difference between the AV-aligned and single-task control conditions (left), and between the AV-aligned and dual-task control conditions (right). Bar to the right indicates scale.

Asking participants to ignore the music and visually monitor for the target word in the single-task control condition caused little change in ASSR relative to the AV-aligned condition (Figure 2, left topographical plot) in either hemisphere (*t* [27] = –0.185, *p*>0.854 for left-frontal ROI and *t* [27] = –0.823, *p*>0.417 for right-frontal ROI). Thus, it is unlikely that a conscious decision to disengage from the music explains the left-lateralized ASSR reduction in the AV-misaligned condition.

The requirement to comprehend and remember the story while also performing the target-word task in the dual-task control condition substantially engaged attentional resources, evidenced by the fact that the response time and accuracy for responding to the target word were significantly degraded in the dual- versus the single-task control condition (response time: 637 ms [s.e.m. = 17] vs. 573 ms [s.e.m. = 12], *t* [27] = 5.373, *p*<0.00002; error rate: 0.167 [s.e.m. = 0.024] vs. 0.078 [s.e.m. = 0.021], *t* [27] = 2.970, *p*<0.007). Despite the fact that the visual reading-comprehension task substantially diverted participants’ attentional resources, ASSR in the dual-task control condition was not reduced relative to the AV-aligned condition in either hemisphere (Figure 2, right topographical plot; *t*[27-0.722, *p*>0.477 for left-frontal ROI and *t*[27-0.648, *p*>0.522 for right-frontal ROI).

The lack of influence of the single- and dual-task control conditions on ASSR reasonably rules out the possibility that the left-lateralized reduction in ASSR in the AV-misaligned condition was due to attentional disengagement from the music. Overall, ASSR from the left-frontal ROI was equivalent in the AV-aligned and the two control conditions, but was selectively reduced in the AV-misaligned condition (Figure 3; confirmed by a significant contrast of {+1, +1, +1, –3} assigned to the four conditions, *t* [27] = 3.533, *p*<.0016, *d* = 0.67).

## Discussion

We tested the hypothesis that left-lateralized auditory mechanisms, proposed by some to process rapidly varying features in sounds, might also contribute to the tracking of the dynamic alignment of auditory and visual features. To test this hypothesis in a naturalistic context, we used music (Beethoven’s *Moonlight Sonata*) and a professionally designed visualizer (iTunes *Jelly*) that presented variations in the luminance, color, motion, and organization of visual features concurrently with variations in the loudness and pitch of the music. To identify an electrophysiological correlate of tracking auditory-visual dynamic alignment over and above stimulus-specific responses, we freshly generated a visualizer display on each trial for each participant; thus, both the AV-aligned and AV-misaligned conditions contained different varieties of visual features except that the displays were synchronized with the music in the AV-aligned condition and desynchronized with the music in the AV-misaligned condition. To monitor auditory sensory responses to the music, we amplitude-modulated the music at 40-Hz and recorded ASSR phase-locked to the modulation, while our visualizer displays (presented on a 60-Hz monitor) could not directly contribute to ASSR.

Compared with when the visualizer displays were temporally aligned with the music, ASSR from the left-frontal (but not right-frontal) ROI was reduced when the visualizers were temporally misaligned relative to the music. There were no significant changes in the degree of phase-locking (ITPC) to the 40-Hz modulation or in the ongoing (non-stimulus-locked) oscillatory neural activity, suggesting that a dynamically misaligned complex visual display reduces the amplitude of left-lateralized auditory responses to music without measurably influencing the temporal fidelity of auditory responses or non-sensory responses, including those associated with cognition, emotion, or arousal.

The control conditions revealed that neither consciously ignoring the music, performing a visual task, nor substantially engaging attentional resources in a concurrent reading-comprehension task caused any reduction in ASSR. Some studies have reported that the level of auditory engagement modulates ASSR [35]–[37]. However, as previously pointed out by de Jong *et al.* [38], in those studies auditory engagement was directed toward the specific amplitude-modulation frequency that produced ASSR by using a modulation-rate discrimination task. Thus, our results along with those of others [38], [39] suggest that ASSR is relatively insensitive to the level of auditory engagement unless the listener selectively attends to the specific amplitude-modulation rate that produces ASSR. Our control results in particular suggest that the reduced left-frontal ASSR in the AV-misaligned condition was unlikely to have been caused by attentional disengagement from the music.

The usual approach to investigating the mechanisms encoding auditory-visual dynamics has been to compare neural activity across conditions in which auditory and visual dynamics are similar but deviate subtly from synchronization [14], [31], [49]–[51]. We know of no other studies making the present type of comparison, distinguishing the effect of temporal misalignment between dynamically comparable auditory and visual streams (the AV-aligned vs. AV-misaligned conditions), from the effect of clear dynamic incongruence between auditory and visual streams (the AV-aligned condition vs. the single- and dual-task control conditions). This distinction is important because auditory and visual dynamics are clearly incongruent much of the time in the real world. For instance, we may look at a computer screen while hearing a truck back up, watch someone walk a dog while listening to a friend talking on the phone, or perform daily tasks while listening to music. Our results may suggest that left-lateralized auditory mechanisms ignore auditory-visual dynamic misalignment in these cases when auditory and visual streams have clearly dissimilar dynamics and we do not notice or pay attention to the sensory misalignment. In contrast, when auditory and visual dynamics are similar, they are likely to originate from the same source, and if so, the redundant cross-modal signals facilitate the accurate processing of the sensory information arising from that source. Yet our natural sensory environment is often abuzz with multiple auditory and visual streams sharing similar dynamics, such as multiple people walking and talking as flying insects buzz around, creating similar limb motions and footsteps, lip movements and speech sounds, and flying paths and amplitude/frequency modulated buzzing sounds. In such a busy sensory environment, binding a visual stream to its corresponding auditory counterpart may require sensitivity to temporal alignment between dynamically similar auditory and visual streams. We speculate that left-lateralized auditory mechanisms that reduce activity when auditory-visual dynamics are similar but misaligned may contribute to correct auditory-visual binding by reducing activity when a false auditory-visual pair happens to be attended. It is possible that these mechanisms also play a part in generating the odd sensation we experience when watching a poorly dubbed movie.

In summary, our results are consistent with the idea that left-lateralized auditory cortical mechanisms continuously track complex dynamic alignment between visual and auditory streams, but only when auditory and visual dynamics are similar, making the processing of dynamic auditory-visual alignment particularly useful for cross-modal binding in a busy sensory environment. The results are also consistent with the idea that the left auditory cortical specialization for the processing of rapidly varying features of sounds (for review, see [29]) extend to the processing of complex auditory-visual dynamic congruence. Our use of naturalistic auditory-visual stimuli that included multiple features varying across multiple time scales, while providing some ecological validity to our neural correlate of monitoring auditory-visual dynamic synchrony, precluded systematic analyses of ASSR relative to the dynamic relationships among the specific auditory and visual features. Future research is necessary to understand how the left-lateralized mechanisms respond to different cross-modal feature combinations (auditory loudness and pitch, vs. visual luminance, color, size, motion, and organization) and to different time scales in which dynamic alignment and misalignment occur.

## Supporting Information

Figure S1 Histogram of the difference in the visual-luminance-vs.-auditory-intensity correlation (*r*) between the AV-aligned and AV-misaligned conditions for 1000 randomly sampled 60-s segments of music (see text for details).
(TIF)

Text S1 Visualizer control experiments.
(DOCX)
