# Supplementary figures and images for: Seeing the Song: Left Auditory Structures May Track Auditory-Visual Dynamic Alignment
Source: PLoS One. 2013 Oct 23;8(10):e77201. doi: 10.1371/journal.pone.0077201 (PMC3806747; doi:10.1371/journal.pone.0077201)

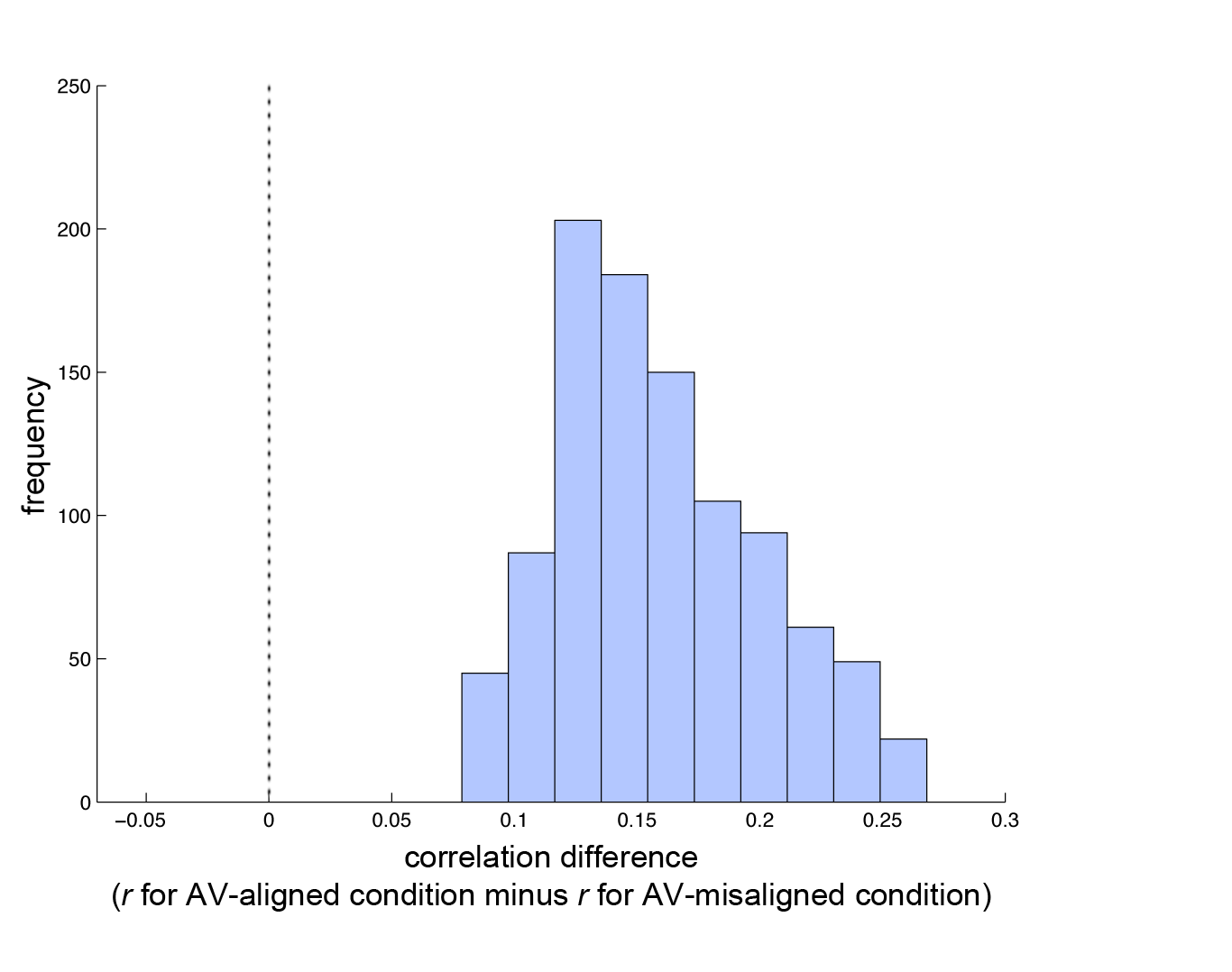

Supplement: Figure S1 — Histogram of the difference in the visual-luminance-vs.-auditory-intensity correlation (r) between the AV-aligned and AV-misaligned conditions for 1000 randomly sampled 60-s segments of music (see text for details). (TIF) [file pone.0077201.s001.tif]
