# Supplementary material for: Seeing the Song: Left Auditory Structures May Track Auditory-Visual Dynamic Alignment
Source: PLoS One. 2013 Oct 23;8(10):e77201. doi: 10.1371/journal.pone.0077201 (PMC3806747; doi:10.1371/journal.pone.0077201)
Supplement: Text S1 — Visualizer control experiments. (DOCX) [file pone.0077201.s002.docx]

**Supplementary Information**

*Visualizer control experiments*

Because *iTunes* Jelly is proprietary visualizer software, we had no access to the algorithm used to create the dynamic images. To verify that the visualizer display was synchronized with the music in the AV-aligned condition, we assessed the synchronization of the visualizer display to the music using perceptual and stimulus-based methods.

*Perceptual assessment of synchronization between the visualizer display and the music.* Twenty naive participants aged 18-29 (13 females), who did not participate in any other condition, were asked to judge the relative synchrony of the auditory and visual streams in the AV-aligned and the AV-misaligned conditions (see *Methods*, main text). We presented the two conditions as they were presented in the main experiment with presentation order counterbalanced across participants. We asked the participants to identify the AV-aligned condition in which auditory and visual streams were synchronous. Seventeen of the 20 participants were able to correctly identify the AV-aligned condition (*p*<0.0003, binomial test). As new visualizer displays were used for each condition for each participant (as in the main experiment), this result indicates that perceived auditory-visual synchrony was greater in the AV-aligned than AV-misaligned condition, over and above variations in stimulus features from display to display. Upon debriefing, the most common observation among the participants who correctly identified the AV-aligned condition was that they felt the luminance of the visualizer display covaried with the intensity profile of the music. Thus, as a means to verify auditory-visual synchrony in an objective, stimulus-based manner, we computed the temporal correlation between the overall luminance variation in the visual display and the overall intensity variation in the music.

*Stimulus-based assessment of synchronization between the visualizer display and the music.* Based on studies on visual detection (see [1] for a review), and visual motion perception (*e.g.,* [2,3]), we estimated that a high end of the visual sampling rate is about 15 Hz. We therefore averaged both visual luminance (summed over all pixels) and auditory intensity into 1/15-s bins. To statistically compare the luminance-vs.-intensity temporal correlation between the AV-aligned and AV-misaligned conditions, we randomly sampled six non-contiguous 10-s intervals from the 120-s music track, assuming that participants would attend to the stimuli about half the time (60 s total) and in about 10-s stretches. Sampling details, however, do not affect the overall correlation result. We computed the average temporal correlation between the six music segments and the corresponding segments (for the AV-aligned condition) and the 30-s shifted segment (for the AV-misaligned condition) from the visualizer display. We then computed the difference in this correlation between the AV-aligned and AV-misaligned conditions with positive values indicating that the visual luminance and auditory intensity are more strongly correlated in the AV-aligned than AV-misaligned condition. We computed this correlation difference 1000 times, each time randomly sampling the six intervals, and the result is presented as a histogram in Figure S1. It is clear that the correlation difference is positive in all cases.

Thus, using both perceptual and stimulus-based methods we have verified that at least for the relationship between visual luminance and auditory intensity, the auditory-visual dynamics were more synchronous in the AV-aligned condition than in the AV-misaligned condition.

**References**

1. Mathewson KE, Lleras A, Beck DM, Fabiani M, Ro T, et al. (2011) Pulsed out of awareness: EEG alpha oscillations represent a pulsed-inhibition of ongoing cortical processing. Front Psychol 2: 99.

2. Simpson WA, Shahani U, Manahilov V (2005) Illusory percepts of moving patterns due to discrete temporal sampling. Neurosci Lett 375: 23-27.

3. VanRullen R, Reddy L, Koch C (2005) Attention-driven discrete sampling of motion perception. Proc Natl Acad Sci U S A 102: 5291-5296.
